# Rapid Quantitative Determination of Enantiomeric Excess by Vibrational Circular Dichroism Using Noise Based Spectral Selection

**DOI:** 10.1002/chir.70134

**Published:** 2026-08-18

**Authors:** Amy M. Jackson, John R. Bows, Geoff R. Nash

**Affiliations:** ^1^ Natural Sciences, Faculty of Environment, Science and Economy University of Exeter Exeter UK; ^2^ PepsiCo R&D Leicester UK

**Keywords:** adulteration, chiral, essential oils, infrared, limonene, multivariate analysis, SNR, VCD

## Abstract

Vibrational circular dichroism (VCD) spectroscopy enables direct determination of enantiomeric excess (ee), but its application as a quantitative analytical tool is limited by low signal‐to‐noise ratios (SNR), which typically require long measurement times. Using limonene as a model system, predictive models were developed by combining targeted, SNR‐based region selection with simple multivariate regression applied to both 30‐ and 5‐min acquisition data. The 30‐min data yielded excellent model performance with cross‐validated *R* ^2^ of 0.997 and RMSE of ~2% ee. Notably, the 5‐min acquisition retained ~97% of predictive performance enabling a sixfold increase in throughput, albeit with increased prediction error. This work therefore demonstrates that data‐driven optimisation of spectral selection can enable reliable ee prediction with short acquisition VCD spectra, outperforming equivalent univariate models and providing a practical route toward high‐throughput chiral analysis.

## Graphical abstract

Rapid VCD‐based determination of enantiomeric excess in limonene is achieved using signal‐to‐noise guided spectral selection and multivariate modeling. While 30‐min spectra provide high accuracy, 5‐min acquisitions retain ~97% performance, enabling faster throughput and offering a practical approach for rapid, reliable chiral analysis in applications such as essential oil authentication.

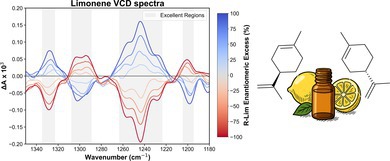

## 1 Introduction

Monoterpenes like limonene (C_10_H_16_) are among the most abundant volatile constituents in the human diet. They are the major components of essential oils found in the oleoresins of various plants including citrus fruits and conifers. Limonene is widely used as a fragrance and flavoring agent and serves as an important synthetic material [1]. It is listed as generally recognized as safe (GRAS) in the Code of Federal Regulation [2] and is commonly incorporated as an additive in foods and cosmetic products. Like most monoterpenes, limonene is chiral, existing as two non‐superimposable mirror image enantiomers with distinct sensory and biological properties. The R‐enantiomer, which predominates in nature, is typically associated with a pleasant, citrusy scent, while the S‐enantiomer is described as having a harsher, more turpentine‐like scent [3]. Beyond sensory attributes, limonene is also associated with antimicrobial, chemo‐preventive and antioxidant activities [4, 5, 6, 7].

Essential oils are highly vulnerable to adulteration with synthetic materials or lower value oils, both of which can impact the characteristic concentrations of terpene enantiomers. Enantiomeric excess (ee) can therefore serve as a reliable marker for quality assessment and authenticity of essential oils, especially when considered alongside the relative concentrations of other chiral components. Accurate determination of ee is therefore valuable both for authenticity testing and for studies where bioactivity depends on stereochemistry.

Vibrational circular dichroism (VCD) spectroscopy offers direct chiroptical information without derivatisation. VCD measures the differential absorption of right and left circularly polarized light by a chiral sample, largely in the mid‐infrared region, yielding spectra in which pure enantiomers display absorption patterns of equal magnitude but opposite sign. This provides structural information through vibrational transitions and enables correlation between molecular structure and stereochemical properties. The first high‐quality, commercially available FT‐VCD spectrometer, the ChiralIR, was introduced by BioTools in 1997 [8], helping to standardize instrumentation and workflows for routine measurements. Today, VCD spectroscopy is considered a valuable alternative to traditional methods for assessing enantiomeric purity, offering reduced sample preparation requirements and the ability to determine absolute configuration [9]. VCD belongs to the broader family of vibrational optical activity (VOA) techniques, which also includes Raman optical activity (ROA).

VOA has found widespread application in the analysis of monoterpenes as they typically produce high quality spectra and are often readily available as single enantiomers. The VCD spectrum of limonene has been reported in several publications in both the mid and near infrared wavelength regions [10, 11], with one study extending this range to visible regions [12]. These studies show good agreement in absorption bands, although the spectra contain many features, complicating interpretation. To address this, Lipp and Nafie applied Fourier self‐deconvolution to limonene spectra to retrieve information obscured by band overlap in the region 940–1350 cm^−1^ [13]. While limonene's VCD profile is well documented qualitatively, quantitative applications for limonene are limited. More broadly, VOA techniques have shown considerable promise for enantiomeric excess determination. VCD‐based ee determination has been demonstrated for other chiral monoterpenes, including α‐pinene and camphor [14, 15, 16], demonstrating a strong linear dependence between VCD response and enantiomeric composition. Similarly, ROA has been used for quantitative ee analysis, with reported errors as low as 0.05% ee for α‐pinene following spectral normalization and careful spectral region selection [17]. However, achieving this level of precision required acquisition times of approximately 5–6 h per sample, highlighting the trade‐off between analytical sensitivity and measurement speed.

Despite these previous successes, noise level remains the limiting factor for quantitative analysis. VCD signals are typically several orders of magnitude smaller than their corresponding infrared (IR) absorbances. In IR measurements the noise level for a given optical setup is dependent on measurement time [8]. Therefore, standard VCD practice has relied on long acquisition times, up to several hours [18], to achieve adequate signal‐to‐noise ratios for small differential absorbances. This temporal constraint has limited the adoption of VCD to in‐line analysis of authenticity, where rapid feedback is essential for real‐time process control. Previous attempts to accelerate VCD have focused primarily on instrumental improvements including the employment of quantum cascade lasers as a high‐power, narrowband mid‐IR source [19]. However, optimisation of data analysis workflows remains underexplored. Recent advances in multivariate analysis have demonstrated significant performance improvements in other areas of vibrational spectroscopy [20, 21], paving the way for its application to VCD quantitative analysis.

In this study, we show that careful band selection can identify mid‐IR features with strong chiroptical contrast and minimal spectral interference, allowing reliable ee estimates to be made with shorter measurement times. Using limonene as a model molecule, we demonstrate that accurate ee determination can be achieved via multivariate modeling with acquisition times as short as 5 min, representing a significant step toward rapid, high‐throughput implementation of VCD for chiral analysis.

## 2 Materials and Methods

### 2.1 Sample Preparation and Spectral Acquisition

R‐(+)‐limonene (97%) and S‐(−)‐limonene (96%) were purchased from commercial suppliers and used without further purification. Enantiomeric excess (ee) standards ranging from −100% to +100% were prepared volumetrically by mixing appropriate volumes of pure enantiomers in increments of 25% ee, calculated according to Equation (1):

(1) $$\mathrm{ee}=\frac{\left[R\right]-\left[S\right]}{\left[R\right]+\left[S\right]}\cdot 100\%$$

where [R] and [S] represent the volumes of R‐ and S‐ limonene enantiomers respectively. Here, ee is defined relative to R‐limonene such that +100% ee corresponds to pure R‐(+)‐limonene and −100% ee corresponds to pure S‐(−)‐limonene. Therefore, positive values indicate an excess of the R‐enantiomer, while negative values indicate an excess of the S‐enantiomer.

All samples were analyzed at room temperature, neat, without solvent dilution. Sample mixtures were transferred to a sealed liquid spectrophotometer cell with CaF_2_ windows and a fixed path length of 50 μm.

VCD spectra were collected using the Bruker PMA 50 module equipped with a photoelastic modulator operating at 50 KHz. Spectra were collected in the region 2000–800 cm^−1^ at 4‐cm^−1^ resolution using two different acquisition times: 30 and 5 min. All measurements were performed in duplicate to assess reproducibility and allow statistical validation of the analytical method. Corresponding FTIR spectra were recorded for each VCD measurement to confirm sample integrity. FTIR absorptions demonstrated identical spectral profiles across all enantiomeric compositions, verifying that observed VCD differences were attributable to chirality.

### 2.2 Spectral Processing and Signal Analysis

Baseline correction was applied using racemic limonene (0% ee) as the reference to eliminate systematic instrumental artifacts and drift effects. Spectra then underwent Savitsky–Golay smoothing with a window length of 13 and third order polynomial fitting to reduce noise while preserving spectral features. Signal‐to‐noise ratio (SNR) analysis was performed across the entire spectral range to identify optimal regions for quantitative analysis. The VCD signal at each wavenumber was calculated as the average absolute signal of the pure enantiomers (Equation 2), while the noise levels were determined from the standard deviation of replicate measurements (Equation 3).

(2) $$\begin{array}{c} \text{Signal}=\frac{\left|{\mathrm{VCD}}_{+100\%}|+|{\mathrm{VCD}}_{-100\%}\right|}{2} \end{array}$$

(3) $$\begin{array}{c} \text{Noise}=\sqrt{\frac{\sum_{i}{\left(\mathrm{Rep}1_{i}-\mathrm{Rep}2_{i}\right)}^{2}}{2N}} \end{array}$$

where Rep1 and Rep2 represent duplicate measurements across all ee concentrations and *N* is the total number of data points.

Excellent spectral regions suitable for multivariate modeling were identified using automated selection criteria based on signal quality metrics. Regions were required to have SNR ≥ 5.0, with a minimum width of 2 cm^−1^, and to be separated from other regions by at least 4 cm^−1^. Selected regions were subjected to sign‐preserved trapezoidal integration to extract peak areas to help mitigate the effects of small shifts in peak position. Defined excellent regions were then used to build the predictive model.

### 2.3 Model Development and Performance Evaluation

Linear regression models were constructed using individual replicates (*n* = 16). The multivariate approach utilized integrated areas from all identified excellent regions as input features so that each sample was characterized by multiple spectroscopic signatures simultaneously. Ordinary least squares (OLS) linear regression was employed to establish quantitative relationships between integrated spectral features and known ee values. To assess the suitability of the multivariate approach, univariate models were developed in parallel using traditional peak height regression from the single highest SNR peak in each dataset. Model performance was evaluated using a fivefold cross‐validation (CV) procedure; multiple complementary metrics were considered to ensure robust assessment of analytical capability. The cross‐validated coefficient of determination (CV‐*R* ^2^) quantified the proportion of variance explained by the model, while the cross‐validated root mean square error (CV‐RMSE) provided a direct measure of prediction accuracy in units of % ee. Limits of detection (LOD) were also calculated to provide practically relevant metrics based on model uncertainty, where LOD = 3 × CV‐RMSE. Performance retention between the two acquisition times was quantified as the ratio of 5‐ to 30‐min CV‐*R* ^2^ values, enabling assessment of throughput optimization potential.

## 3 Results and Discussion

**FIGURE 1:**
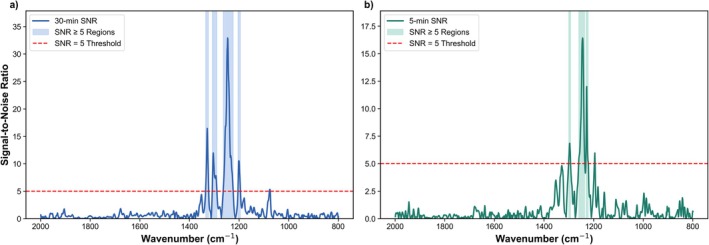
Signal‐to‐noise ratio (SNR) analysis of limonene VCD spectra: (a) 30‐ and (b) 5‐min acquisition times. The dashed red line indicates the SNR = 5 threshold. Shaded areas highlight the spectral regions with an SNR ≥ 5 that were selected for the multivariate analysis.

This VCD‐based methodology for rapid enantiomeric excess (ee) determination of limonene shows strong potential for practical chiral analysis applications. Figure 1 shows SNR analysis across the whole measured spectral range. The stringent SNR ≥ 5 threshold ensured selection of only the most analytically robust spectral regions, which were then partitioned into distinct regions by identifying gaps greater than 4 cm^−1^. A final geometric filter, requiring a minimum width of 2 cm^−1^, aided elimination of noise artifacts and ensured selection of coherent molecular vibrational features for analysis. Small apparent SNR features are also observed in regions where little or no VCD signal is expected. These are attributed to residual instrumental noise and measurement variability rather than genuine chiroptical bands. As a result, these regions do not satisfy the SNR threshold used for selection of informative spectral features.

For the 30‐min acquisition data (Figure 1a), four regions met the selection criteria, and were classified as ‘excellent regions,’ whereas three such regions were identified for the 5‐min dataset (Figure 1b). The 30‐min dataset also exhibited superior SNR characteristics with maximum values reaching 32.9 and averaging 10.7 across excellent regions. Meanwhile the 5‐min data achieved maximum SNR values of 16.4, averaging 8.1 across excellent regions. Notably, both datasets identified similar spectral regions as optimal for analysis, primarily in the 1350‐ to 1180‐cm^−1^ range, corresponding to characteristic C‐O and C‐C stretching vibrations in the limonene structure.

**FIGURE 2:**
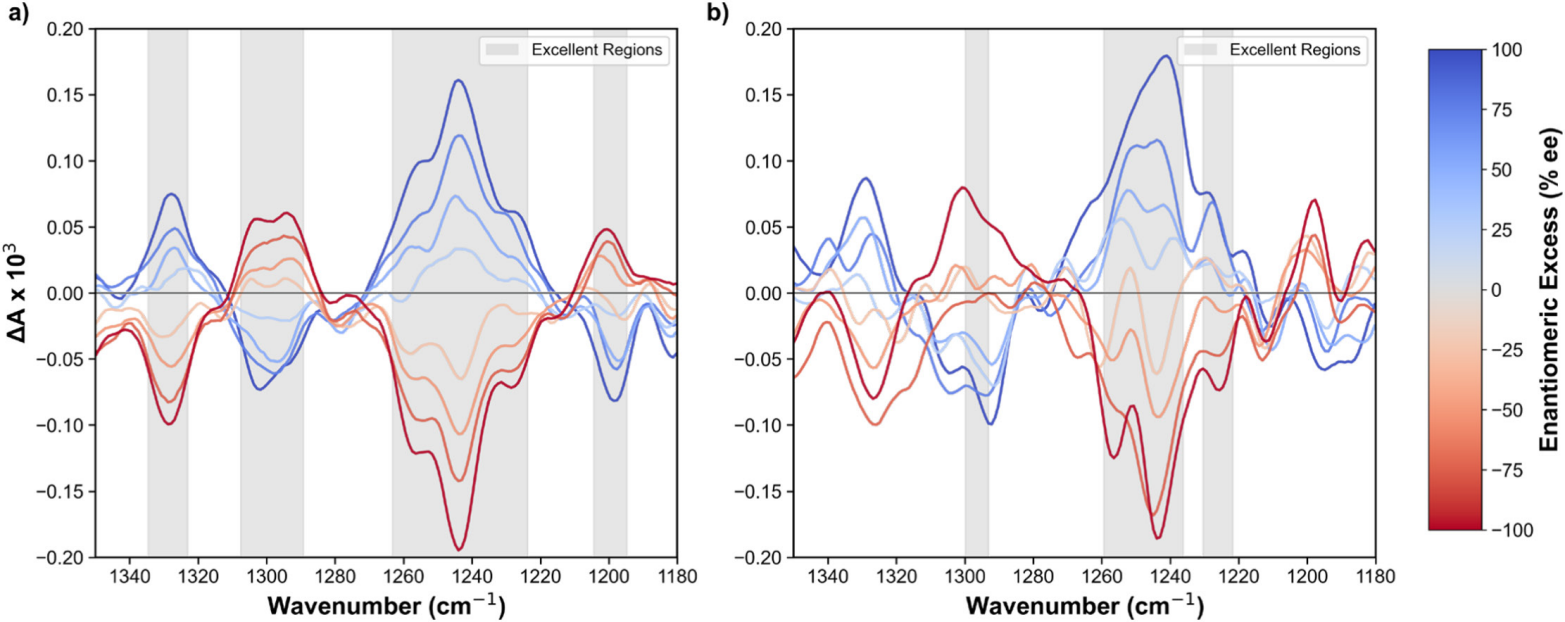
Preprocessed VCD spectra of limonene. VCD spectra of limonene for (a) 30‐ and (b) 5‐min acquisition times over the 1350‐ to 1180‐cm^−1^ spectral range. Individual spectra are colored according to enantiomeric excess (% ee), defined relative to R‐limonene (+100% = pure R, −100% = pure S), as indicated by the color bar. The gray shaded areas highlight “Excellent Regions” identified from signal‐to‐noise ratio analysis (Figure 1) used for quantitative model development.

The baseline corrected and smoothed 30‐ and 5‐min VCD spectra of the different limonene enantiomeric mixtures in this range are shown in Figure 2a,b, respectively. Baseline correction and Savitzky–Golay smoothing successfully reduced noise while preserving essential spectral features, as evidenced by the clean spectra and consistent enantiomeric patterns observed across all concentration levels, especially for the 30‐min dataset. As expected, VCD signals of the enantiomers are equal and opposite, with their magnitude proportional to % ee. This ‘mirror image’ relationship is visually evident across the 1150‐ to 1350‐cm^−1^ region; however, outside of this spectral range features appeared noisy or inconsistent. The excellent regions identified through SNR analysis align well with the most pronounced VCD features, validating the effectiveness of the SNR‐based region selection approach.

**FIGURE 3:**
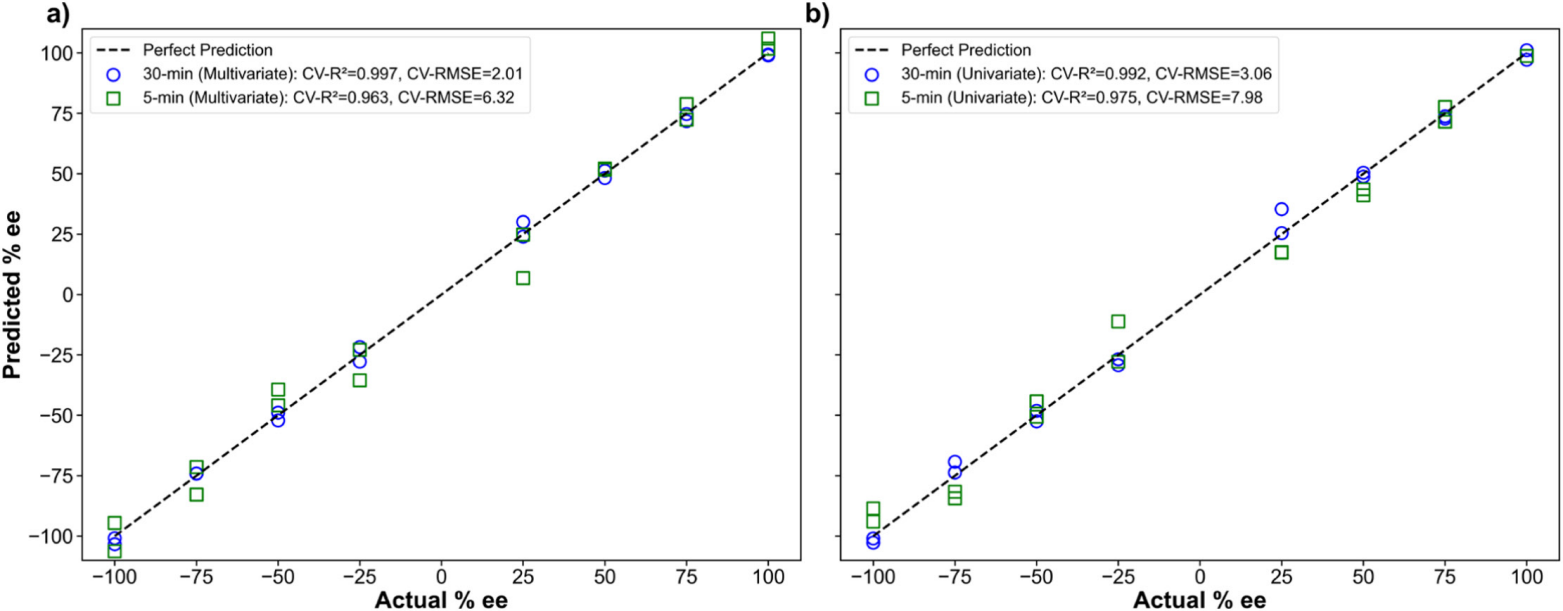
Regression models for limonene quantitation. Regression plots of actual vs. model‐predicted enantiomeric excess (% ee) for (a) the multivariate model and (b) the univariate model. Both plots display results from the 30‐min (blue circles) and 5‐min (green squares) spectral acquisitions. Model performance metrics were determined using fivefold cross‐validation and are displayed in the legend. The dashed line in each plot represents a perfect prediction (*y* = *x*).

The multivariate and univariate linear regression models built from the integrated areas of all selected regions and single peak intensities respectively were used to evaluate quantitative performance across both acquisition time data sets. Cross‐validated performance metrics and corresponding regression plots are presented in Table 1 and Figure 3.

**TABLE 1:** Summary of fivefold cross‐validated performance metrics. Results are shown for both multivariate and univariate calibration models at 5‐ and 30‐min acquisition times. CV‐*R* ^2^ = cross‐validated coefficient of determination; CV‐RMSE = cross‐validated root mean square error; LOD = limit of detection. Feature count refers to the number of spectral regions included in each model.

Method	CV‐*R* ^2^	CV‐RMSE	LOD	Features
30‐min multivariate	0.996534	2.01%	6.0%	4
5‐min multivariate	0.962803	6.32%	19.0%	3
30‐min univariate	0.991576	3.06%	9.2%	1
5‐min univariate	0.975094	7.98%	23.9%	1

For 30‐min measurements, the model achieved strong performance with CV‐*R* ^2^ = 0.9965, CV‐RMSE = 2.01% ee, and LOD = 6% ee. This LOD signifies the model's ability to reliably distinguish a sample from a pure racemic mixture (0% ee), indicating its sensitivity to small chiral imbalances. While the 30‐min acquisition provides robust quantitative performance, the key objective of this work was to investigate whether reliable ee determination could be maintained at substantially shorter acquisition times. Crucially, the 5‐min multivariate model maintained strong predictive performance despite a sixfold reduction in acquisition time, with CV‐*R* ^2^ = 0.9628 and CV‐RMSE = 6.32% ee. Although prediction error increased relative to the 30‐min model, overall performance remained robust and within acceptable limits for qualitative analysis. This level of accuracy is often sufficient for authenticity screening applications, such as verifying the chiral integrity of essential oils where the primary goal is to detect significant deviations in enantiomeric composition indicative of adulteration rather than to precisely resolve small variations [22].

Regression plots (Figure 3) illustrate good linearity across all models with slightly increased scatter around the ideal prediction line observed for shorter acquisition times. Univariate models (Table 1, Figure 3b) were built using only the single highest SNR peak from each dataset to contextualize the benefit of the multivariate approach. While univariate models exhibited linearity, their performance was consistently inferior to the corresponding multivariate approach. Multivariate modeling reduced CV‐RMSE by factors of ~1.5 for the 30‐min dataset and ~1.3 for the 5‐min dataset relative to univariate analysis. As a result, LOD improvements were even more pronounced, demonstrating that reliance on a single peak, historically common in VCD quantitation, limits robustness and sensitivity, particularly in noisy or time‐constrained measurements. The multi‐region integration strategy therefore represents a significant methodological improvement, leveraging the distributed chiroptical signatures of the molecule rather than over‐weighting a single peak that may be susceptible to noise or minor spectral shifts.

The consistency between the 30‐ and 5‐min datasets validates the method's robustness with respect to acquisition time. The 5‐min method retains ~96.6% of the predictive performance of the 30‐min method, indicating only a modest reduction in explained variance despite a sixfold reduction in acquisition time. However, this gain in throughput is accompanied by increased prediction error and higher limits of detection, highlighting a trade‐off between speed and sensitivity. The 30‐min acquisition protocol already represents a substantial improvement over conventional VCD timescales, providing excellent analytical performance in a fraction of the time typically required. The 5‐min protocol further pushes the boundaries, enabling near real‐time VCD analysis suitable for process monitoring and high‐throughput screening. These results demonstrate that VCD spectroscopy, when coupled with targeted region selection and multivariate analysis, offers a rapid, non‐destructive alternative to traditional chromatographic methods for enantiomeric excess determination, with significantly reduced analysis times suited to process monitoring and quality control applications.

## 4 Conclusions

This study demonstrates that VCD, when combined with objective SNR‐based region selection and multivariate modeling, enables rapid and reliable determination of limonene enantiomeric excess. By integrating information across multiple high‐quality spectral regions, the multivariate approach substantially outperformed traditional univariate, single‐peak analysis in predictive accuracy, as reflected by improved *R* ^2^ and reduced RMSE. The 30‐min acquisition protocol provided excellent quantitative performance (RMSE ≈ 2% ee), representing a significant improvement over conventional multihour VCD measurements. Notably, the 5‐min protocol retained ~96.6% of the predictive power of the 30‐min measurement while reducing acquisition time sixfold, highlighting the feasibility of rapid VCD analysis even under low SNR conditions.

These findings address long‐standing limitations of VCD as a quantitative tool and demonstrate that data‐driven spectral region selection can unlock substantial gains in throughput while maintaining robust predictive capability. Compared with approaches that rely on regression of manually selected VCD bands, the present workflow combines objective SNR‐guided spectral region selection with multivariate modeling to utilize information from multiple informative spectral regions simultaneously. This provides a more systematic framework for quantitative enantiomeric excess determination, particularly for short‐acquisition spectra. The automated region‐selection workflow also reduces operator bias and increases method reproducibility, enhancing its suitability for routine deployment.

Beyond limonene, the methodology is readily transferable to other chiral terpenes and small molecules that exhibit distributed chiroptical responses across the mid‐infrared spectrum. While limonene provides an ideal benchmark due to its relatively strong VCD response, future studies should evaluate the methodology using more challenging chiral systems that exhibit weaker chiroptical signals, greater conformational flexibility, or substantial spectral overlap. Such systems will provide a more stringent assessment of the robustness and general applicability of the proposed SNR‐guided workflow. With further validation across broader molecular classes, this approach shows strong potential to support high‐throughput authenticity screening and rapid quality control. The data‐driven nature of the proposed workflow may also enable its adaptation to other spectroscopic regions, such as the important near‐infrared region, although this remains to be explored in future studies. Future work could also explore the integration of machine learning methods with the established SNR‐guided region selection to further enhance performance by capturing subtle, non‐linear spectral relationships. Such developments may facilitate quantitative deconvolution of overlapping chiroptical signals in multicomponent systems and could push the boundaries of rapid chiral analysis toward even shorter acquisition times while maintaining analytical precision.

## Data Availability

The data that support the findings of this study are available from the corresponding author upon reasonable request.
